# Deciphering Key Interactions of Ligands with CYP3A4-Template* system

**DOI:** 10.14252/foodsafetyfscj.D-20-00023

**Published:** 2021-02-10

**Authors:** Yasushi Yamazoe, Takashi Yamada, Akihiko Hirose, Norie Murayama

**Affiliations:** 1 Division of Drug Metabolism and Molecular Toxicology, Graduate School of Pharmaceutical Sciences, Tohoku University, 6-3 Aramaki-Aoba, Aoba-ku, Sendai 980-8578, Japan.; 2 Division of Risk Assessment, National Institute of Health Sciences, Tonomachi 3-25-26, Kawasaki-ku, Kawasaki 210-9501, Japan; 3 Laboratory of Drug Metabolism and Pharmacokinetics, Showa Pharmaceutical University, Machida, Tokyo194-8543, Japan.

**Keywords:** Keywordsthalidomide, lenalidomide, ebastine, tocopherols, BHT, acetaminophen

## Abstract

Cytochrome P450 (CYP)-mediated metabolisms are often associated with biological and toxicological events of chemicals. A major hepatic enzyme, CYP3A4, showed clear distinctions on their catalyses even among ligands having resemble structures. To better understand mechanisms of their distinct catalyses, possible associations of ligand interactions at specific parts of CYP3A4 residues were investigated using CYP3A4-Template system developed (DMPK 2019 and 2020). A placement was available selectively for CYP3A4-mediated R-thalidomide 5-oxidation on Template, but not for the 5’-oxidation and the S-isomer oxidations. Similar placements were generated for pomalidomide (4-amino-thalidomide), but not for a poor ligand, lenalidomide (3-deoxy-pomalidomide). The latter ligand took placements lacking IJK-Interaction or sticking the 4-amino part beyond the facial-side wall on Template. A placement was available for the *tert*-butyl oxidation of terfenadine, but not for an analog, ebastine. Their interactions with upper-Cavity-2 residue were expected to differ at their sites of oxygen substituents. Some phenolic antioxidants behave distinctly toward biological oxidations *in vitro* and *in vivo*. Butylated hydroxytoluene is oxidized to the peroxy-derivative *in vitro*, but solely to the oxidized metabolites at the benzyl and *tert*-butyl methyl positions *in vivo*. Involvement of CYP3A4 were suggested for all the three reactions from the placements on Template. Tocopherols were also applied on Template for the oxidations for chroman and side-chain terminals. The primary placement was suggested to undergo the futile-recycling through formation of the peroxide intermediate subsequently to lead the substantial lack of the CYP3A4-mediated oxidation. These data suggest the effectiveness of CYP3A4-Template assessment to understand the causal basis of poor oxidations and also to verify the *in vivo* contribution of CYP3A4-mediated peroxidative reactions.

## 1. Introduction

Cytochrome P450 family of enzymes (CYP) catalyze oxidations and reductions of hydrophobic substances including steroids, fatty acids and industrial chemicals. CYP enzymes belonging to CYP1-4 families are involved in the metabolism of xenobiotics and exhibit distinct catalytic-properties with each other. Their substrate specificities overlap among individual enzymes to some extents. These CYP enzymes, however, exhibit distinct catalyses toward slight changes in ligand structures. These phenomena have been known with various ligands, and explained mostly as altered interactions of whole ligand-molecules with the whole active sites of CYP enzymes, except for heme ligands. No detailed mechanisms for their distinct catalyses have been characterized for hydrophobic ligands. Currently, 3D-protein models derived from crystalized ligand-bound CYP3A4 are available to study the ligand interactions^1^^,^^2^^)^. The clear verification of good and poor substrates is, however, yet to be available for the general ligands.

Human CYP enzymes are in unique situations, of which substrate specificities are investigated in details with the use of the recombinant enzyme systems. The resultant data of their catalyses have been accumulated for more than twenty-years. With uses of the advantages, we reconstituted the active sites of the CYP enzymes through assemblies of ligands as fused-grid Templates^3^^,^^4^^,^^5^^,^^6^^,^^7^^)^. These Template systems have been refined with introductions of ideas of region-specific interactions and step-wise movements in the active site such as Right-side movement. For examples, ligands interactions of CYP1A1 (>350 reactions)^8^^)^, CYP1A2 (>450 reactions)^9^^)^ and CYP3A4 (>1,100 reactions)^10^^)^ were reproduced faithfully with more than 99% accuracies of their regioselectivity and stereoselectivity. The failures (inconsistency) are due to the secondary phenomena like NIH-shifts and nonenzymatic cyclizations of intermediates in these reactions.

Metabolisms of drugs and chemicals are often associated heavily with subsequent events such as pharmacological efficacies and toxicological outcomes. Prediction of the metabolism with deciphering information is expected to support the safety assessments.

To understand the causes of distinct CYP3A4-mediated metabolisms between closely related ligands, typical good- and poor-ligand pairs are applied on CYP3A4 Template system in the present study. CYP3A4 mediates *in vitro* peroxidative reactions of various chemicals. The role of these peroxidative reactions in the body is still in dispute. Thus, possible mechanisms of distinct roles of CYP3A4-mediated peroxidative reactions between *in vivo* and *in vitro* are also discussed.

## 2. Materials and Methods

Experimental information on the substrate specificities and metabolites on CYP3A substrates was obtained from literatures. The published data on recombinant human CYP3A4, CYP3A5 and CYP3A7 systems were used preferably because of the direct reflections of the catalytic properties of individual CYP3A enzymes. Chem3D (version 5 for Mac OS, CambridgeSoft, Cambridge, MA) and ChemBio3D (version 12 for Windows, CambridgeSoft) and ChemBioDraw (versions 11 and 13 for Mac OS, CambridgeSoft/PerkinElmer) were used to construct two-dimentional (2D) or three-dimensional (3D) structures of the substrates and to overlay compounds on Template. Several template-terms are defined to explain ligand interactions with Template in our previous studies^10^^,^^11^^,^^12^^,^^13^^,^^14^^,^^15^^)^. These terms are listed in a separate section as “Template terms used”.

Substrates of CYP3A enzymes, except for polyaromatic hydrocarbons, take various conformations due to their flexibility. Prior to the Template application, chemicals are taken in their flattened form(s). The flatted or extended shapes of 3D structures were tried to sit on Template and then modified their conformations to fit within Template in consideration of the bond-energy barrier using MM2 function of Chem3D and specific interaction at distinct regions of Template. Carbon, oxygen, nitrogen, sulfur and halogen atoms of 3D ligand structures are indicated with gray, red, blue, yellow and green symbols, respectively. The hydrogen atoms of the substrates were not considered for the placement.

Templates consist of hexagonal grids and sticks. The sitting of substrate atoms at each corner of the hexagonal-grids (termed Rings) was evaluated as occupancy. An atom that could not be placed exactly at the corner was evaluated as located at the closest corner. The placement of substrates in text is expressed in a hyphen-linked form, such as Rings A-B-C, to trace the occupancy of chemical molecules on Template. CYP3A4 ligands are assumed to migrate from Entrance to Site of oxidation without changing the conformation. Thus, ligands enter as the same conformations as observed at the Site of oxidation.

Chemicals including lactone moieties are often ionized at neutral pH ranges. These lactones were treated as ionizable groups for the application of substrates. Thus, sittings of non-rigid lactone rings are not allowed at Rings B, D, E, K and L, unless otherwise the presence of stabilizing influence^11^^)^.

CYP3A4 ligands need to fulfill three essential contacts at Site of oxidation (Position 6), Trigger-site (Position 26) and at least a part of Rings I, J, K for IJK-Interaction. These ligands interact with Template by ways of uni-molecule and bi-molecule bindings. In cases of bi-molecule binding, ligands sitting at Site of oxidation are termed pro-metabolized molecules. The molecules are necessary to occupy both Site of oxidation and at least a part of Rings I-J-K region for I-J-K-Interaction. Another molecule termed trigger molecule occupies Trigger-site (Position 26). Both pro-metabolized and trigger molecules are required to stay simultaneously within Width-gauge. All the three sites are occupied with single molecule in uni-molecule binding. Pro-metabolized molecule and trigger molecule need to have a slight overlapping point(s) on Template, although trigger molecules are situated behind to contribute for the immobilization of pro-metabolized molecules. To escape contacts with heme-oxygen atom, trigger molecules do not occupy Positions 5, 6 and 7 on Template. The occupancy of Trigger-site (Position 26) happens with trigger molecule in bi-molecule binding or with a part of a ligand in uni-molecule binding. Cavity-2 residue descended would immobilize ligands to trigger the catalyses in the Template system. Other experimental details of CYP3A4, CYP3A5 and CYP3A7 Template systems are described in our publications^10^^,^^11^^,^^12^^,^^13^^,^^14^^,^^15^^)^.

## 3. Results

### 3.1 Placements of Thalidomide, Pomalidomide and Lenalidomide

Thalidomide is a teratogenic agent. The possible association of the metabolism has been discussed on the thalidomide-induced teratogenicity^16^^)^. CYP3A4 mediates the 5-oxidation of R-thalidomide, but not of S-thalidomide^17^^)^. No 5’-oxidation of thalidomide is detected with CYP3A4. This enzyme catalyzes the 5-oxidation and slightly the 7-oxidation of pomalidomide (4-amino-thalidomide)^18^^)^. CYP3A4, however, does not mediate substantially the ring-oxidation of lenalidomide (3-deoxy-pomalidomide)^19^^,^^20^^)^.

**Fig. 1.**
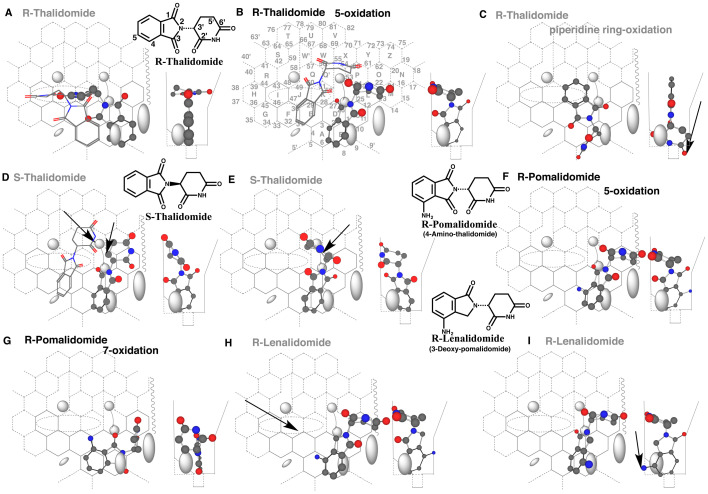
Interactions of thalidomide, pomalidomide and lenalidomide on Template Placements of R-thalidomide for the 5-oxidation (A and B) and for 5’-oxidation (C), of S-thalidomide for 5-oxidation (D and E), of R-pomalidomide for the 5- (F) and 7-oxidations (G), and of R-lenalidomide for the 5-oxidation (H and I) are shown as their 3D structures. Ligands at Site of oxidation and at a gate between Bay-1 and upper-Cavity-2 residues are shown in cylindrical- and stick-shapes, respectively. 2D-Structures of R- and S-thalidomide, R-pomalidomide and R-lenalidomide are shown near the 3D-structures. Arrows indicate the possible causes of defects. Functional and non-functional placements are distinguished with dark and gray colors of structure names, respectively.

CYP3A4 ligands enter Template from the left side (Rings G, H, R and S, Fig. 1)^13^^)^. R-Thalidomide was thus expected to move to the right direction at least in two distinct conformations, the phthalimide part in the front or the bottom, in considering the thickness allowance indicated with Width-gauge (Fig. 1 right side). A placement for the 5-oxidation of R-thalidomide was constructed at Rings A-E(F)-K-J-I as pro-metabolized molecule of bi-molecule binding (Fig. 1A stick-shape). The molecule passed a gate between Bay-1 and upper-Cavity-2 residues^13^^)^ and moved further to the right direction after reaching to Ring A. The movement would lead the phthalimide part to pass through the rear side of Front-residue (Fig. 1A cylindrical-shape). The Right-side movement resulted in the absence of R-thalidomide part at Site of oxidation. Only thin ligands pass at the facial-side of Cavity-2 residue, if not hit to upper-Cavity-2 residue^13^^)^. A molecule of R-thalidomide, generated by 180° rotation of the molecule shown in Fig. 1A, hit to upper-Cavity-2 residue during the pass at the gate between Bay-1 and upper-Cavity-2 residues and did not reach to Site of oxidation (Data not shown).

Another placement for the 5-oxidation was available at Rings A-B-C(D-K)-L-M (Fig. 1B cylindrical-shape). This molecule managed to pass a gate between Bay-1 and upper-Cavity-2 residues, through migrating above of upper-Cavity-2 residue (Fig. 1B stick-shape). The migration was terminated by the contact with Front-residue. The placement fulfilled minimally the interaction at Rings I, R and/or K for IJK-Interaction, in addition to two other essential contacts at Site of oxidation and Trigger-site (Position 26). The regioselective oxidation of this ligand at the 5-position was suggested from the sitting around Position 6 of Template.

A placement for the possible 5’-oxidation of R-thalidomide was constructed at Rings A-B-D(C)-K plus Position 6’ (Fig. 1C). The 6’-carbonyl part was, however, unable to sit within Groove, and thus the placement was not available.

S-Thalidomide was also expected to pass the gate between Bay-1 and upper-Cavity-2 residues. Two distinct placements were constructed for the 5-oxidation at Rings B-D-C-L-P-O plus Cavity-2 (Fig. 1D) and at Rings A-B-D-C-L plus Cavity-2 (Fig. 1E). The invasions of the 2’,6’-glutarimide part into Cavity-2 region were, however, not allowed on both the placements^11^^)^. In addition, the phthalimide part stayed at Ring B was unable to interact with heme-oxygen (Fig. 1D). Thus, differences in configurations of the 2’,6’-glutarimide ring of R- and S-thalidomide were linked with the descending allowance of Cavity-2-residue to trigger at Position 26.

A placement of R-pomalidomide was available at Rings A(E)-B-C(D-K)-L-M (Fig. 1F). Similar to the placement of 5-oxidation of R-thalidomide, the oxidation of R-pomalidomide was expected to occur at the 5-position from the molecule sitting at Ring A. Another placement of R-pomalidomide was generated at Rings A(E)-B-C-L (Fig. 1G). The 1,3-dioxopyrrolidine part managed to pass the gate to reach to Site of oxidation. The 3-keto part on 1,3-dioxopyrrolidine ring stayed at Position 26 served for triggering and the 4-amino group contributed minimally the IJK-Interaction for the 7-oxidation.

In a way similar with R-thalidomide and R-pomalidomide, a conformation taking phthalimide part in the bottom was expected for an approach of R-lenalidomide into Template. A placement for the 5-oxidation of R-lenalidomide were constructed at Rings B(A)-D-C-L-M (Fig. 1H). The placement, however, had no contact for IJK-Interaction. Another placement of R-lenalidomide was constructed at Rings B-C(D)-L-M (Fig. 1I) in similar ways to the 5-oxidation of R-thalidomide and R-pomalidomide (Figs. 1B and F). This placement was also nonfunctional due to the 4-amino-group exceeding at the facial-side wall of Width-gauge. Results from placements of thalidomide, pomalidomide and lenalidomide on CYP3A4 Template were consistent with experimental data of their CYP3A4-mediated oxidations.

### 3.2 Placements of Terfenadine and Ebastine

**Fig. 2.**
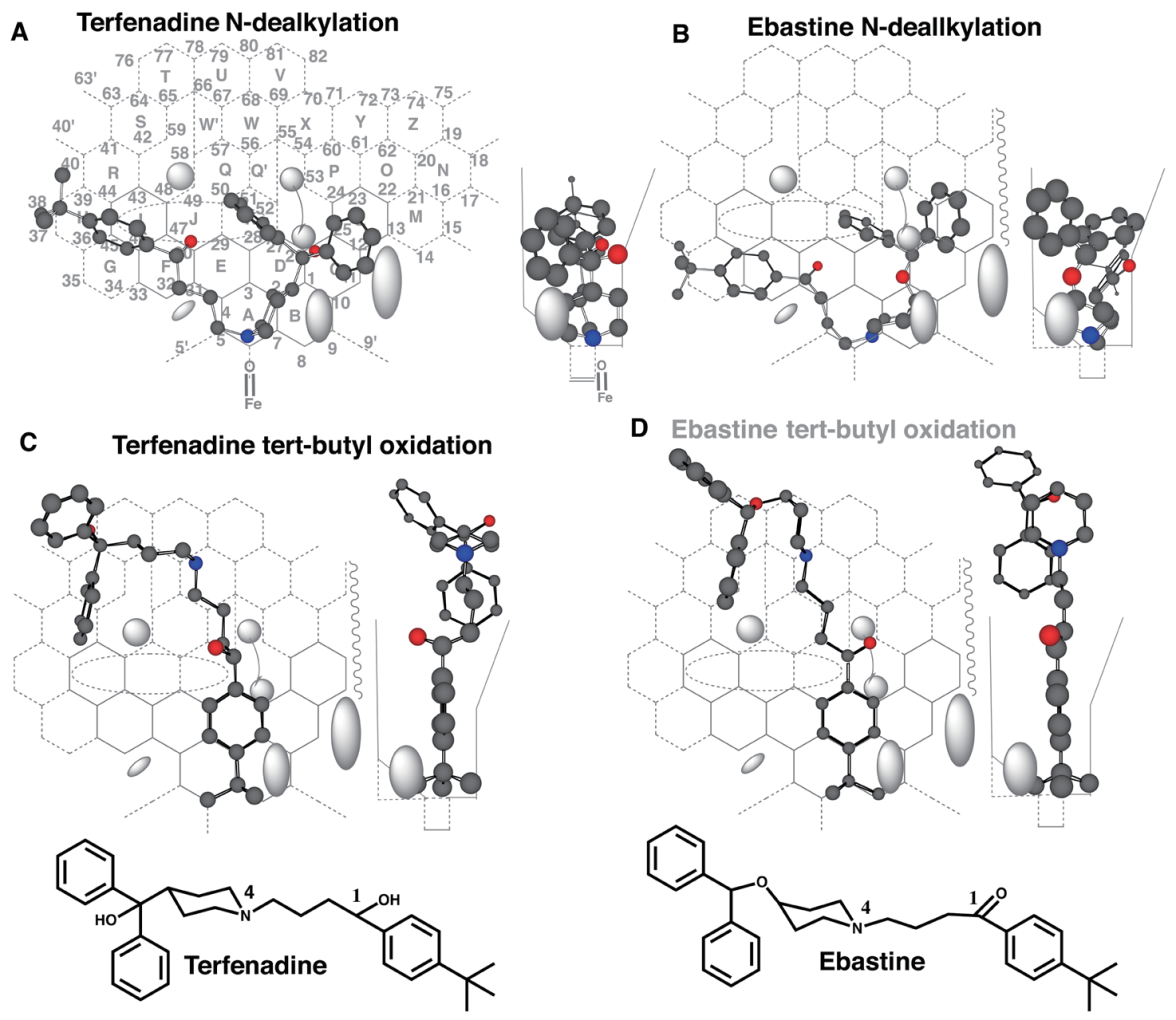
CYP3A4-mediated oxidations of terfenadine and ebastine and their placements. Placements for the *N*-dealkylations of terfenadine (A) and ebastine (B), and for *tert*-butyl oxidations of terfenadine (C) and ebastine (D) are shown as the 3D structures. 2D-Structures of terfenadine and ebastine are also shown with parts of position numbers. Functional and non-functional placements are distinguished with dark and gray colors of structure names.

Terfenadine and ebastine are antagonists of the histamine H_1_ receptor. Terfenadine used as prodrug was superseded by the active metabolite, fexofenadine, due to the risk of cardiac arrhythmia of the parent drug. Both terfenadine and ebastine resemble in 2D-structure with each other (Fig. 2). CYP3A4 mediates *N*-dealkylations of both terfenadine and ebastine. This P450 mediates also the *tert*-butyl oxidation of terfenadine, but not of ebastine.

A placement for the *N*-dealkylation of terfenadine was available at rings H-I-F(J)-E-A-B-D(K)-C-L as uni-molecule binding (Fig. 2A, only the 1R-form is shown). A phenyl part of diphenylmethane moiety stayed at facial-side and thus passed a gate between Bay-1 and upper-Cavity-2 residues. A similar sitting of ebastine for the *N*-dealkylation was available at Rings G-F-E-A-B-D(K)-C-L (Fig. 2B). The piperidine ring was expected to lean on Front-residue, and the diphenylmethane part thus passed through facial-side of upper-Cavity-2 residue.

A placement for *tert*-butyl oxidation of 1R-terfenadine was available at Rings A(B)-D-K-Q’-W-U-T-S-R plus Position 63’ as uni-molecule binding (Fig. 2C). 1S-Terfenadine was able to take the similar placement (Data not shown). CYP3A4 was thus expected to mediate the oxidation of methyl part of *tert*-butyl group of both R- and S-terfenadines.

Ebastine also took a similar placement at Rings A(B)-D-K-Q’-W-U-T-S plus above of Ring T (Fig. 2D). In this placement, the 1-carbonyl oxygen atom would interfere the descending of upper-Cavity-2 residue to Trigger site (Position 26). Attempts of facial-side shift of the 1-carbonyl oxygen atom resulted in the exceeding of the diphenylmethane part out of Width-gauge or of ceiling part. These results suggested the causal basis of the lack of the oxidation of methyl part of *tert*-butyl group of ebastine.

The *tert*-butyl oxidation product of terfenadine, fexofenadine, was only marginally *N*-dealkylated in the body^21^^)^. The conformational change through the zwitterion formation may be associated with the poor oxidation to form the azacyclonol metabolite through the *N*-dealkylation^21^^)^ (Data not shown).

### 3.3 Placement of Butylated Hydroxytoluene (BHT)

BHT is metabolized in the body through oxidations of the benzyl and *tert*-butyl parts to yield two distinct alcohols^22^^,^^23^^,^^24^^)^. In addition, 4-hydroperoxy derivative of BHT and quinone methide are identified *in vitro* and these are proposed to link to the pulmonary toxicity in mice^25^^,^^26^^)^. The formation of the peroxy-derivative is inhibited by carbon monoxide and SKF525-A, suggesting the P450-mediated production, although no data is available for human CYP enzyme(s) involved in the formation of the peroxy-derivative. The P450-mediated cleavage of *O*-*O*-bond of the peroxy-derivative is observed *in vitro*^27^^)^ and *in vivo*^28^^)^. A study using hepatic microsomes of dexamethasone- or phenobarbital-treated rats suggests an interaction of BHT peroxy-derivative with multiple CYP enzymes including CYP3As^29^^)^. Microsomal oxidations of BHT to form alcohols at benzyl and *tert*-butyl moieties are enhanced in livers of mice pre-treated with phenobarbital^30^^)^.

**Fig. 3.**
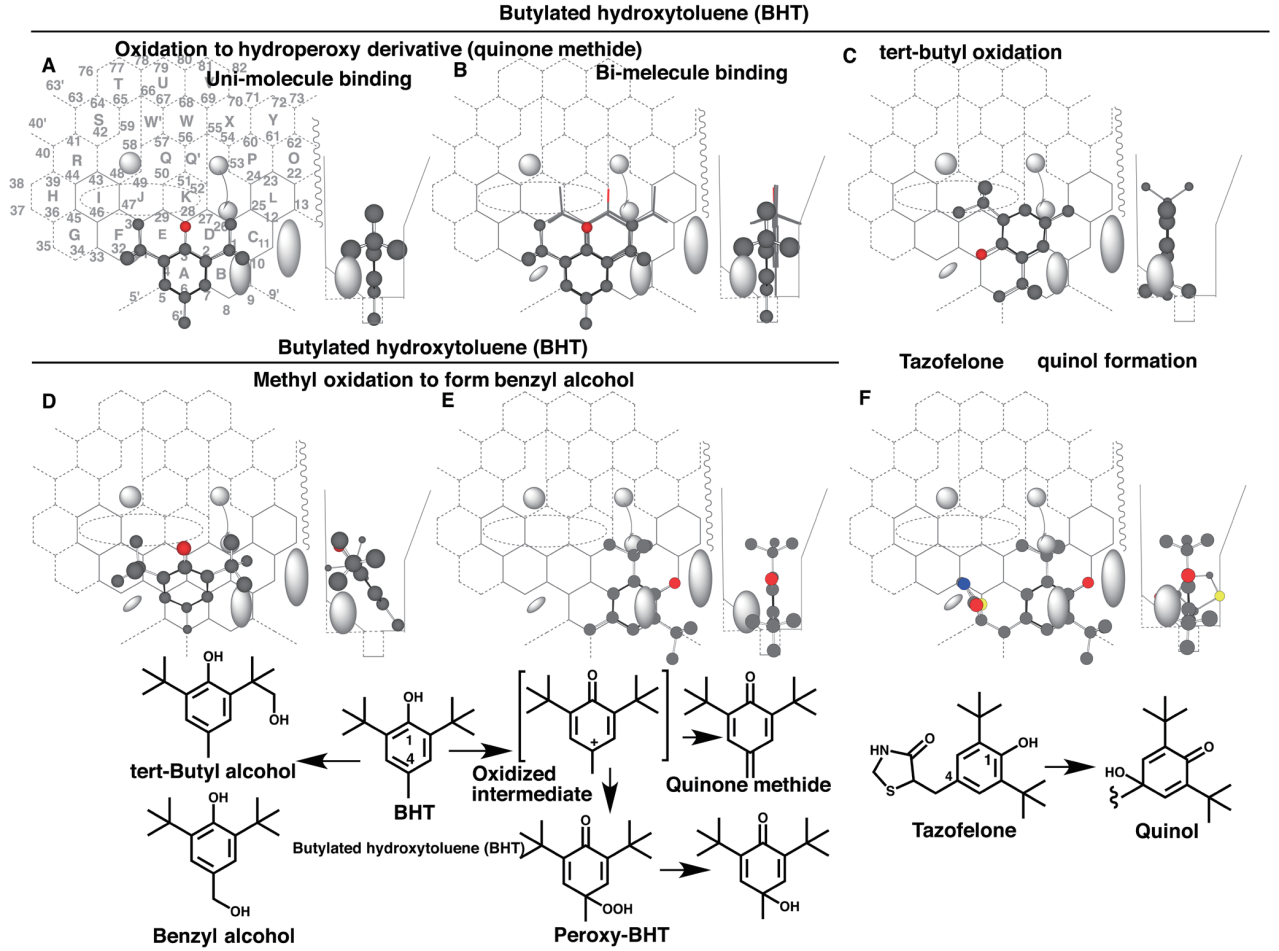
CYP3A4-mediated oxidations of BHT and tazofelone and their placements Placements of BHT for the hydroperoxyl/quinone methide derivatives (A and B), for *tert*-butyl oxidation (C) and methyl oxidation (D and E), and of tazofelone quinol formation (F) are shown as 3D-structures on Template. 2D-Structures of BHT and the metabolites, and also of tazofelone are also shown with parts of position numbers.

Placements for the peroxy-BHT formation was generated at Rings A(D-C)-E-F plus Position 6’ as uni-molecule binding (Fig. 3A) and bi-molecule binding (Fig. 3B). A methyl part of *tert*-butyl group at Position 26 needed to hold Trigger residue in the uni-molecule binding, but might be not stable enough to support the trigger action. Trigger molecule thus might be necessary at Rings B-D(C-L)-K-J (Fig. 3B stick-shape) for the efficient reaction. The bi-molecule binding might be favored for the peroxy-BHT formation due to the exclusive detection of the peroxy-BHT *in vitro* in high substrate concentrations. The peroxy-derivative is substantially undetectable *in vivo*, probably due to the conversion to the alcohol derivative^31^^)^ and reactive quinone methide^27^^)^, and also due to the efficient reduction to parent BHT in cells^32^^)^.

Two placements were constructed for the *tert*-butyl oxidation of BHT at Rings A-B-D(E)-C-L (Fig. 3C) and a flipped placement at Rings A-B-D(C)-D-K-J (Data not shown). Both the BHT molecule entered from Entrance (left side of Template) and migrated at facial-side to reach to Rings A. The former molecule would migrate the *tert*-butyl part to Rings A and B. The *tert*-butyl part of the latter molecule hanged at upper-Cavity-2 residue, although few portions of the latter molecule might pass through the facial side of upper-Cavity-2 residue.

A placement for the benzyl alcohol formation was available at Rings A(D-C)-E-F (Fig. 3D). The *tert*-butyl group contacted with both facial-side wall and Front-residue, and the methyl group with rear-side wall. The methyl part of *tert*-butyl group was thus fixed at Position 26 and the methyl group at Position 6 interacted with heme-oxygen, although the IJK-Interaction was minimal at Ring J. A distinct placement for the benzyl alcohol formation was generated at Rings B(A)-C-D plus Position 9’ (Fig. 3E). As described in a recent study^13^^)^, narrow spaces around Positions 5’ and 9’ are available for ligands. The sitting of a *tert*-butyl part at Position 9’ would afford a sitting for 4-methyl group at Position 6 on Template.

CYP3A4 mediates a quinol formation of tazofelone, a drug having 2,6-di-*tert*-butyl phenol structure^33^^)^. A placement for the quinol formation was available at Rings A(E)-B-C-D plus Position 9’ (Fig. 3F). The initial oxidation at the benzyl part would result in the formation of the 4-hydroxy quinol after the electron delocalization.

Formation rates of quinone methides were higher with the *tert*-butyl alcohol metabolite of BHT than with BHT itself^30^^)^. Increased extents of IJK-Interaction with the dimethyl-hydroxymethyl part might associate with higher rates of the formation of quinone methide through an intermediate placement mutual also for peroxy-BHT formation (Figs. 3A and B). The simulation data described above were consistent with experimental observations and suggested the involvement of CYP3A4 on hepatic oxidation pathways of BHT.

### 3.4 Placements of Tocopherols

In mammals, α-tocopherol, β-tocopherol and γ-tocopherol are major components of vitamin E. Liver is a major site of tocopherol metabolisms. Their metabolism is started by CYP-mediated ω-oxidations. Initially a role of CYP3A enzyme was proposed on the ω-oxidation from the effective inhibition in the presence of ketoconazole or sesamin^34^^)^, but recombinant CYP3A4 and CYP3A7 failed to produce the metabolite of α-tocopherol^35^^)^. CYP4F2 is shown to mediate mainly the ω-oxidations of tocopherols in livers^35^^,^^36^^)^. Higher tissue concentrations on non-α-tocopherols was observed in null mice of cyp4f14, murine orthologue of human CYP4F2, but the concentration of α-tocopherol remained unchanged in general in the null mice^37^^)^. Thus evidence for the involvement of CYP4F2 in vitamin E metabolism is convincing, but the participation of other CYP enzymes such as CYP3A4 cannot be excluded yet on the metabolisms of tocopherols^38^^)^.

**Fig. 4.**
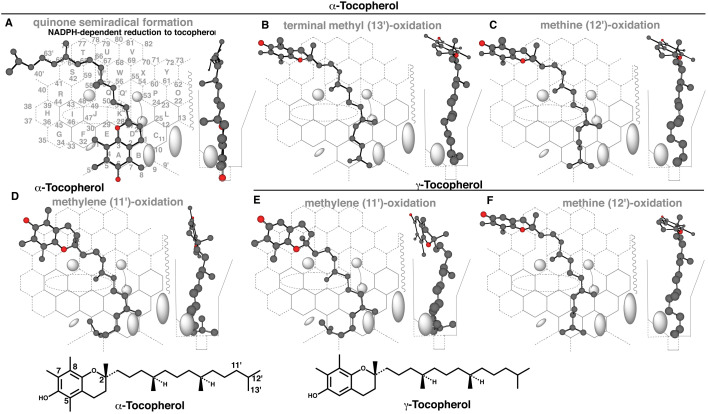
Placements of tocopherols Placements for possible chromanol (A) and side-chain (B, C and D) oxidations of α-tocopherol and side-chain oxidations of γ-tocopherol (E and F) are show as 3D-structures. 2D-Structures of α-tocopherol and γ-tocopherol are also shown with parts of position numbers.

A placement of α-tocopherol as uni-molecule binding was available at Rings A(B)-D(E)-K-Q-W’-T-S plus Positions 5’ and 6’ and Positions 40’ and 63’ of Entrance (Fig. 4A). Sitting of the phenol group at Position 6’ suggested the formation of a quinone semiradical, which would be reduced back to α-tocopherol in the presence of NADPH. Another placement of α-tocopherol was constructed for the ω-oxidation (13’) at Rings A(B)-D-K-Q-W’-T-S plus Position 63’ (Fig. 4B). In addition, placements for the ω-1 (12’) and ω-2 (11’) oxidations were generated at Rings A-D-K-Q-W’-T-S and Position 63’ (Fig. 4C also shown as γ-tocopherol 4F) and at Rings A-B-C-D-K-Q-W’-T-S-R plus Position 40’ (Fig. 4D also shown as γ-tocopherol 4E), respectively. Therefore, CYP3A4-mediated side-chain oxidations of tocopherols were possible, but the futile recycling of the primary placement (Fig. 4A) was likely to diminish or substantially negate the interactions of tocopherols for side-chain oxidations.

### 3.5 Influence of Caffeine on CYP3A4-mediated Acetaminophen Activation

Acetaminophen is metabolized primarily through sulfate and glucuronide conjugations in the body. Oxidations of this chemical to the 3-hydroxy derivative and quinoneimine, so called “NAPQI”^39^^)^, occur in conditions of the high-levels of exposures and/or insufficiency of conjugating capacities. NAPQI is believed to be the reactive intermediate leading the toxicity in liver^40^^)^. Data in humans demonstrate that CYP2E1 is the main enzyme involved in NAPQI production in therapeutic concentrations^41^^,^^42^^)^. Possible involvement of CYP3A4 is also suggested *in vitro* for the NAPQI formation.

CYP3A4-mediated NAPQI formation, as assessed by the glutathione conjugate, was enhanced in the presence of caffeine^43^^)^. Caffeine, but not theophylline, enhanced the formation-clearance of NAPQI in phenobarbital-pretreated rats, providing evidence that activation of CYP-mediated catalysis occurs *in vivo*^44^^)^.

**Fig. 5.**
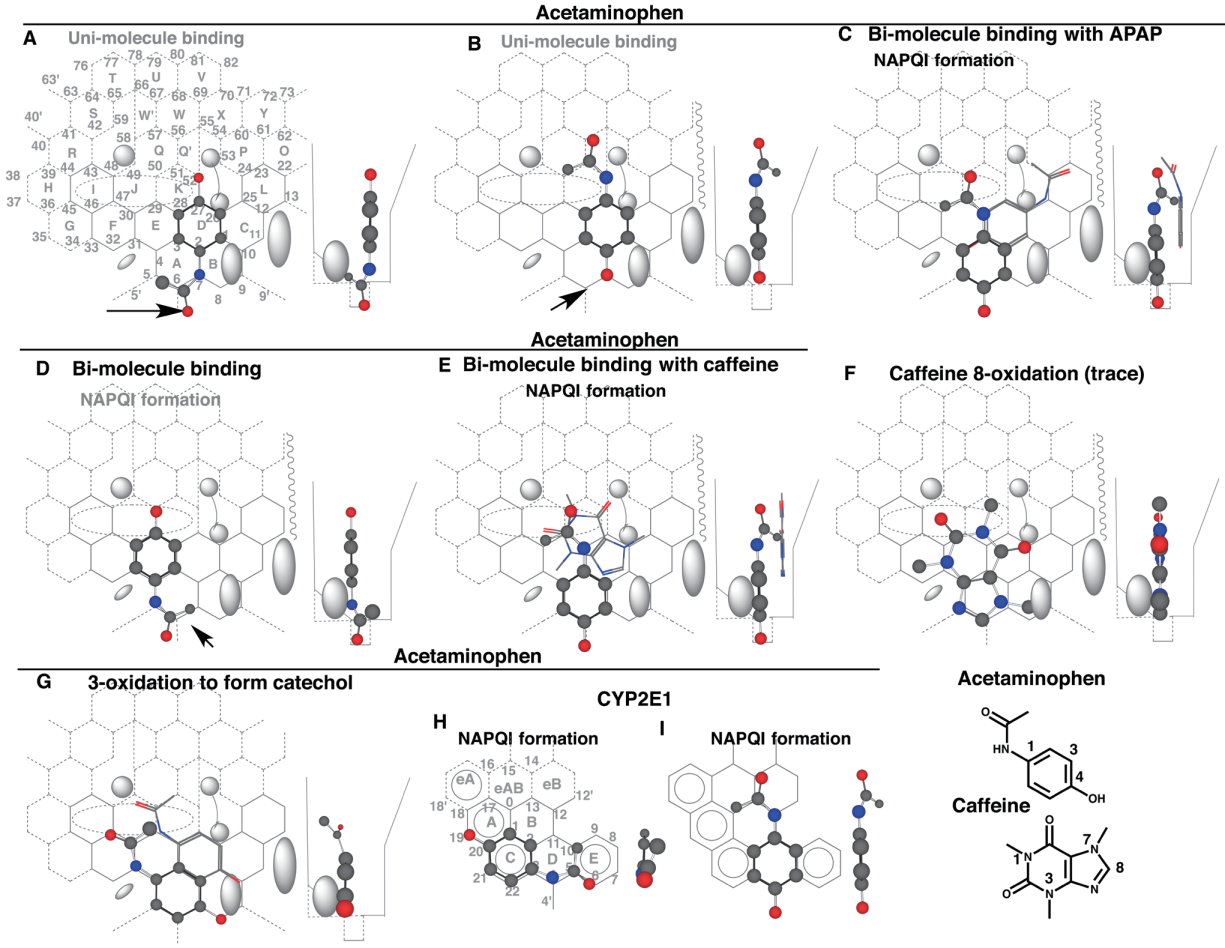
CYP3A4- and CYP2E1-mediated oxidations of acetaminophen and the placements Placements of CYP3A4-mediated acetaminophen oxidations for NAPQI formation as uni-molecule binding (A and B) and bi-molecule binding (C, D and E), of the 8-oxidation of caffeine, of the 3-oxidation of acetaminophen are shown as 3D-structures. Placements of CYP2E1-mediated oxidation for NAPQI formation are also shown in H and I. Trigger molecules are shown as stick-shape in C and E. Trigger molecules are omitted in D and G. 2D-Structures of acetaminophen and caffeine are also shown with parts of position numbers. Functional and non-functional placements are distinguished with dark and gray colors of structure names. Dark arrows indicate possible causes of defects.

Placements of acetaminophen were constructed at Rings A-D-K plus Position 6’ (Fig. 5A) and at Rings D-K-Q’ plus Position 7 (Fig. 5B) in uni-molecule bindings. These molecules needed to fulfill three essential contacts on Template (See Materials and Methods). The acetamide part of the former located in Groove and Position 7 was not oxidized, and 4-hydroxyl part of the latter located at Position 7 was also not oxidized. Another uni-molecule placement was constructed at Rings A-D-C plus Position 5’ for a possible 3-oxidation (Data not shown), but the triggering support with the acetyl part was unlikely to be sufficient. NAPQI formation was thus expected on the bi-molecule placement at Rings A-E-K(J) and Position 6’ for pro-metabolized molecule and at Rings D(E)-C-L(P) for trigger molecule (Fig. 5C). The access of heme-oxygen at 4-position of acetaminophen molecule would lead the quinoneimine (NAPQI) production. Flipping of top and bottom parts of the pro-metabolized molecule generated another pro-metabolized molecule at Rings A-E-J plus Position 5’/6’ (Fig. 5D). The acetyl methyl-oxidation, rather than oxidation of nitrogen atom, was expected from the placement. Caffeine molecule might sit at Rings C-D-E-K-Q(Q’) as trigger molecule (Fig. 5E) in considering the structural difference between caffeine and theophylline (7-*N*-demethylcaffeine) and also sitting avoidance of trigger molecules at Positions 5, 6 and 7. Differing from theophylline, caffeine is a poor substrate of CYP3A4^45^^)^. This chemical might take a placement at Rings B-A-E(C/F)-J-K for the trace level of the 8-oxidation (Fig. 5F). The 7-*N*-methyl part was difficult to sit at Ring B unless otherwise the left-side shift near Bay-1 residue. The poor substrate and poor-inhibitory properties of caffeine to CYP3A4 may also contribute on the enhancing action for NAPQI production in the bi-molecule binding described above, since ligands interacting firmly at trigger-site and without interfering pro-metabolized molecule sittings are feasible for enhancing agents^11^^)^.

A placement of acetaminophen 3-oxidation to form the catechol derivative was available at Rings A(B)-E(F)-J as pro-metabolized molecule. The NAPQI and catechol formations were mediated by bi-molecule bindings of acetaminophen molecules but derived from distinct placements. The NAPQI and catechol formation were thus initiated through the 4- and 3-oxidations of acetaminophen, respectively.

Placements of acetaminophen on CYP2E1 Template^3^^)^ were generated for NAPQI formation at Rings C(A)-D-E (Fig. 5H) and at Rings D-B-eB(eAB) plus Position 4’ (Fig. 5I). Ligands were oxidized at Position of 4 of CYP2E1 Template. The former molecule managed to keep the acetyl group around the bridge part of Rings D and E, and stayed within Template area. This molecule underwent initially the oxidation at the nitrogen atom and then was transformed to NAPQI. The acetaminophen molecule in the latter placement suggested the initial oxidation of the phenol part to form NAPQI. CYP2E1 was thus able to activate acetaminophen through both oxidations of the 1-nitrogen and 4-carbon atoms, whereas CYP3A4 mediated activation only through an oxidation of the 4-carbon atom.

## 4. Discussion

Verification of ligand interactions as good- and poor-substrates and also regioselectivity in their metabolisms are often necessary to evaluate clearly chemical toxicities in humans.

Clear differences in the invasion into Cavity-2 region were detected on placements of R- and S-thalidomide for the 5-oxidation. The glutarimide part of S-thalidomide, but not of R-thalidomide, interfered the descending of Cavity-2 residue to trigger the catalysis at Position 26 (Figs. 1D and E). A placement was constructed for a possible R-thalidomide 5’-oxidation (Fig. 1C). The oxygen atom of the glutarimide part, however, went through the bottom of CYP3A4 Template, if a carbonyl part of the phthalimide was arranged to serve for triggering at Position 26.

Presence of 4-amino group on R-thalidomide structure (R-pomalidomide) had no obvious influence on the 5-oxidation (Fig. 1F), and rather offered the IJK-interaction for a placement of the 7-oxidation (Fig. 1G). Instead, deprivation of the 3-oxygen atom of R-pomalidomide caused defects of IJK-Interaction (Fig. 1H) or of sitting outside of Width-gauge (Fig. 1I). These defects would lead the poor catalysis through CYP3A4-mediated oxidation of R-lenalidomide. The good and poor substrate properties of thalidomide and derivatives were clearly verified through interactions of these isomers at essential regions on Template.

Another type of interference of trigger-residue (upper-Cavity-2 residue)-descending was observed with *tert*-butyl oxidation of ebastine (Fig. 2D). The 1-carbonyl group of terfenadine escaped the hitting with upper-Cavity-2 residue (Fig. 2C), while the 1-hydroxyl group of ebastine contacted with upper-Cavity-2 residue to prevent the descending (Fig. 2D). Similar differences are also observed on the distinct modes of interactions of erythromycin and clarithromycin as described in our previous study^13^^)^. These phenomena, together with interactions of R- and S-thalidomide analogs, supported an essential concept of CYP3A4 Template system, trigger event through descending of upper-Cavity-2 residue (trigger residue).

CYP3A4-mediated peroxidative reactions was also studied to assess the functional significance. Placements of BHT and tocopherols for CYP3A4-mediated peroxidative reactions are constructed on Template. BHT peroxide was detected *in vitro* but tocopherol peroxides were not detected, possibly depending on the reactivities with reducing equivalents in biological systems. CYP3A4-mediated NAPQI formation from acetaminophen (Figs. 5C and E) was also expected to go through the phenoxy radical intermediate.

CYP2E1 rather than CYP3A4 mediates NAPQI formation *in vivo* in humans. Therefore, CYP3A4-mediated peroxidative reactions of all these three phenols are likely to have diminished or trivial contributions *in vivo* in humans. Relatively high levels of reducing equivalent *in vivo* than *in vitro*^46^^)^ may be effective for futile recycling of BHT and tocopherols. Requirement of high substrate concentrations is expected from the selective involvement of bi-molecule binding for CYP3A4-mediated NAPQI formation (Figs. 5C and E). These data suggest the need of cautious evaluation of CYP3A4-mediated peroxidative reactions for toxicological assessments.

## Template Terms Used

**2D and 3D:** two-dimensional and three-dimensional

**Bay 1 and Bay 2:** CYP3A4 residue located lower left and right of Template

**Bi-molecule and Uni-molecule binding:** Interactions on Template with Trigger- and Pro-metabolized molecules combination, and with single molecule

**Cavity-1 and Cavity-2:** Holes in the middle of Template. The residues in the holes (**Cavity-1 residue and Cavity-2 residue**) are expected to participate in the IJK-Interaction and triggering. These residues appear on Template plane after ligand’s passage.

**Front-residue:** Protein residue existing at facial side of Ring B

**Functional and non-functional placements:** Placements linking to metabolite productions or inhibition are functional, and placements without linking to metabolite productions or inhibition are non-functional.

**Futile-sitting:** A phenomenon associated with lack of oxidations of rotatable and non-substituted phenyl group of ligands

**Groove:** A space for ligand sittings located beneath of Width-gauge

**IJK-Interaction:** Interaction of ligands with Rings I, J and/or K region is expected to initiate facial-side movement of ligand

**Pro-metabolized molecule:** Substrates to be oxidized or reduced are termed as “pro-metabolized molecule” in the simulation experiment.

**Right-side movement:** Right-direction shift of ligands entered in Rings A and B to Bay-2 direction

**Trigger molecule:** A molecule, which is not oxidized, acts for triggering the catalysis. Trigger molecules need to have direct contacts to pro-metabolized molecules on 2D Template

**Width-gauge:** A guide tool to judge allowable width for ligand accommodation around Template which was determined empirically mainly with steroid ligands

**Site of Oxidation:** A confined space of enzymatic catalysis. An Area near Position 6 corresponds to Site of Oxidation in CYP3A4 Template

**Trigger-site:** Position 26 of Template, which works to hold Cavity-2 residue and this interaction serves to initiate the catalysis.

## Author contributions

Participated in research design: Yamazoe

Conducted experiments: Murayama and Yamazoe 

Performed data analysis: Yamazoe, Yamada and Hirose

Wrote or contributed to the writing of the manuscript: Yamazoe, Murayama and Yamada
